# Pediatric autoimmune retinopathy and optic neuropathy: a case report and a review of the literature

**DOI:** 10.3389/fopht.2023.1275335

**Published:** 2023-12-15

**Authors:** Hersh Varma, Kevin X. Zhang, Veeral S. Shah

**Affiliations:** ^1^ Department of Ophthalmology and Visual Sciences, The Ohio State University Wexner Medical Center, Columbus, OH, United States; ^2^ Department of Ophthalmology, Nationwide Children’s Hospital, Columbus, OH, United States; ^3^ Abrahamson Pediatric Eye Institute, Division of Pediatric Ophthalmology, Cincinnati Children’s Hospital Medical Center, Cincinnati, OH, United States; ^4^ Medical Scientist Training Program, University of Cincinnati College of Medicine, Cincinnati, OH, United States; ^5^ Department of Ophthalmology, University of Cincinnati College of Medicine, Cincinnati, OH, United States; ^6^ Division of Pediatric Neurology, Cincinnati Children’s Hospital Medical Center, Cincinnati, OH, United States

**Keywords:** autoimmune retinopathy, autoimmune optic neuropathy, cancer-associated retinopathy (CAR), melanoma-associated retinopathy (MAR), autoimmune-related retinopathy and optic neuropathy (ARRON), anti-enolase, anti-recoverin

## Abstract

**Purpose:**

The purpose of the study was to present a rare case of pediatric bilateral optic neuropathy and retinopathy, which was consistent with a diagnosis of autoimmune retinopathy. We also reviewed the most current literature and phenotypes associated with reported pediatric cases of autoimmune retinopathy.

**Design:**

The design of the study was a case report, with a retrospective case series literature review.

**Subjects:**

This study incorporated data from six subjects, with one presenting as an original case report and five being identified from the English-language literature published to date.

**Materials and methods:**

The materials and methods involved a descriptive analysis of fundus findings, electrophysiologic testing, serum autoantibody testing, optical coherence tomography (OCT), brain MRI scanning, and fluorescein angiography, which were performed where available.

**Main outcome measures:**

The study evaluated the clinical presentation and treatment outcomes of all subjects and followed their visual function over time.

**Results:**

All six subjects had retinal abnormalities that were documented on imaging, while five out of the six subjects had optic nerve abnormalities. Electrophysiologic testing was performed on three subjects, all of whom recorded abnormal results. An underlying neoplastic disorder was described for four subjects. Serum autoantibody testing results were available for four subjects. The serum testing included using antibodies against a 22-kDa antigen, a 35-kDa optic nerve-derived antigen, a 62-kDa antigen, enolase, recoverin, tubulin, and pyruvate kinase M2. Our subject presented 12 years after resection of a ganglioglioma with asymmetric bilateral vision loss, disc edema in one eye, advanced disc pallor in the fellow eye, and bilateral subtle retinal infiltrates, despite having a normal fluorescein angiogram. OCT demonstrated asymmetric ganglion cell layer thinning, which is consistent with the vision loss. Our subject also had abnormal brain MRI findings of widespread pachymeningeal enhancement, but he had a normal cerebrospinal fluid composition. He was initially treated with high-dose pulse steroids, followed by intravenous immunoglobulin therapy. He experienced partial visual recovery in both eyes.

**Conclusions:**

Pediatric autoimmune retinopathy and optic neuropathy are rare diseases that can present with unique signs and symptoms. In pediatric patients who present with symptoms of subacute progressive vision loss with negative inflammatory workups, a history of prior neoplasm, and/or clinical findings of progressive retinopathy or optic neuropathy, an autoimmune process should be considered in the differential.

## Introduction

Autoimmune retinopathy and optic neuropathy disorders are a group of heterogeneous clinical entities characterized by acute or subacute bilateral vision and color vision loss, visual field constriction, retinal degeneration, optic nerve swelling, and seropositivity for one or more retinal autoantibodies. These disorders are broadly categorized into paraneoplastic autoimmune retinopathies (pAIRs), such as cancer-associated retinopathy (CAR) and melanoma-associated retinopathy (MAR), and non-paraneoplastic autoimmune retinopathies (npAIRs), which are often associated with systemic autoimmune disorders. Although both pAIRs and npAIRs are most commonly seen in elderly patients, children can be affected as well. In this study, we present a case of pediatric autoimmune retinopathy and optic neuropathy, occurring in a 15-year-old male with a history of resected left temporal ganglioglioma a decade earlier.

## Methods—case presentation

A 15-year-old male, otherwise healthy, presented with a 6-month history of progressive bilateral vision loss. His past medical history included a diagnosis of epilepsy secondary to a left temporal ganglioglioma. He underwent total resection at age 3 years, with no evidence of residual disease during a 3-year surveillance period by oncology. He had no ocular symptoms, abnormal head position, or history of failed vision screening prompting an ocular examination until this presentation.

**Figure 1:**
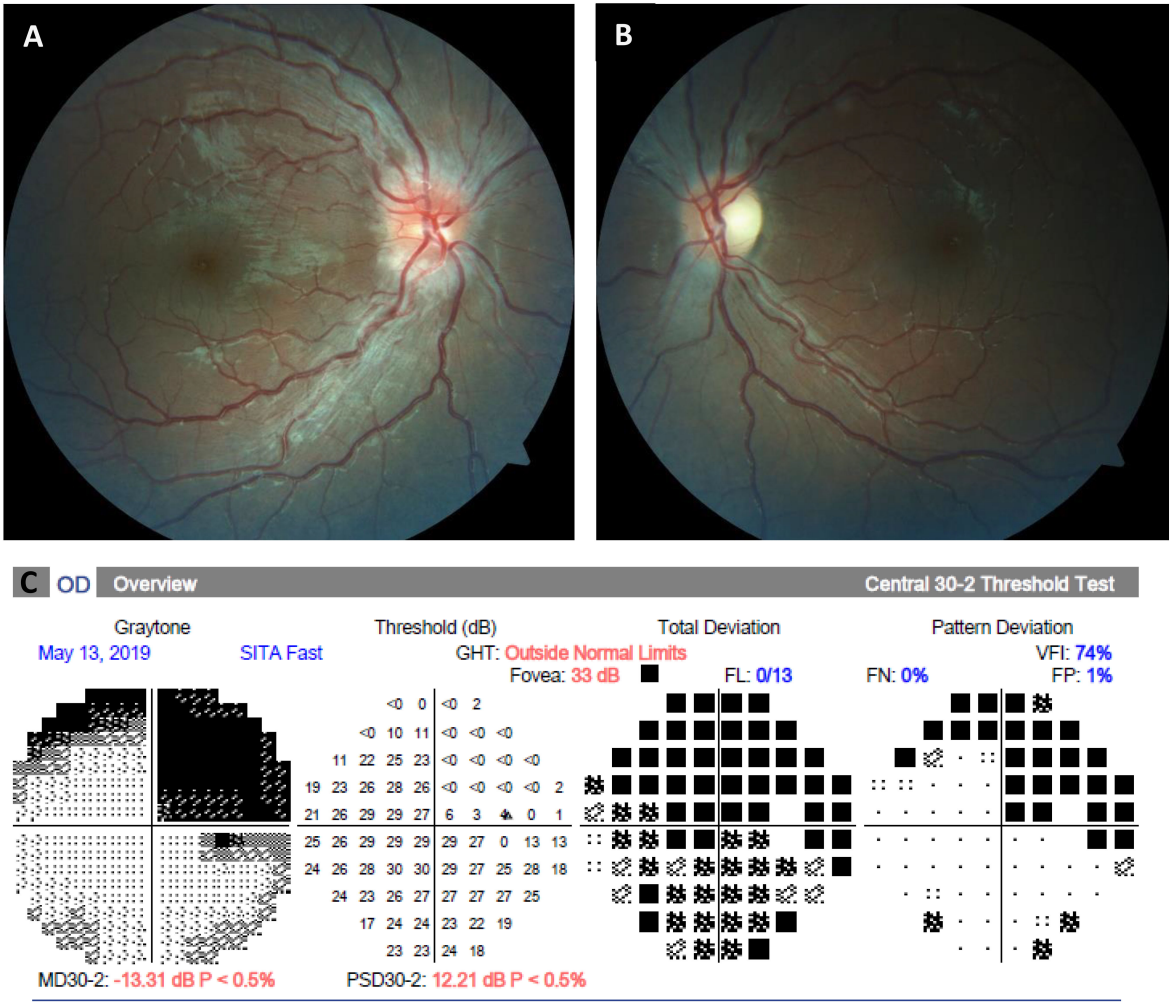
Fundus photographs and automated perimetry at presentation. **(A, B),** Color fundus photographs demonstrating an asymmetric right greater than left optic disc edema. **(C)**, Visual field examination demonstrating right superior quadrantanopia in the right eye, which is presumed to be due to a previous left temporal ganglioglioma resection. The left eye was unable to perform automated perimetry due to decreased vision.

Six months prior to this presentation, he began noticing that he was experiencing vision difficulties in his left eye, but he did not recognize the severity until he began covering his right eye. In addition, he began noticing early similar right eye vision loss and presented with this as his principal complaint. During the initial presentation to the general pediatric ophthalmologist, he noted a visual acuity of 20/20 in the right eye and the ability to detect hand motion in the left eye, with an afferent pupillary defect and diminished color vision. Visual field testing showed that he had right superior temporal quadrantanopia of the right eye, and he was unable to perform in the left eye. A fundus examination revealed moderate disc edema in the right eye and mild edema in the left eye (**Figure 1**).

**Figure 2:**
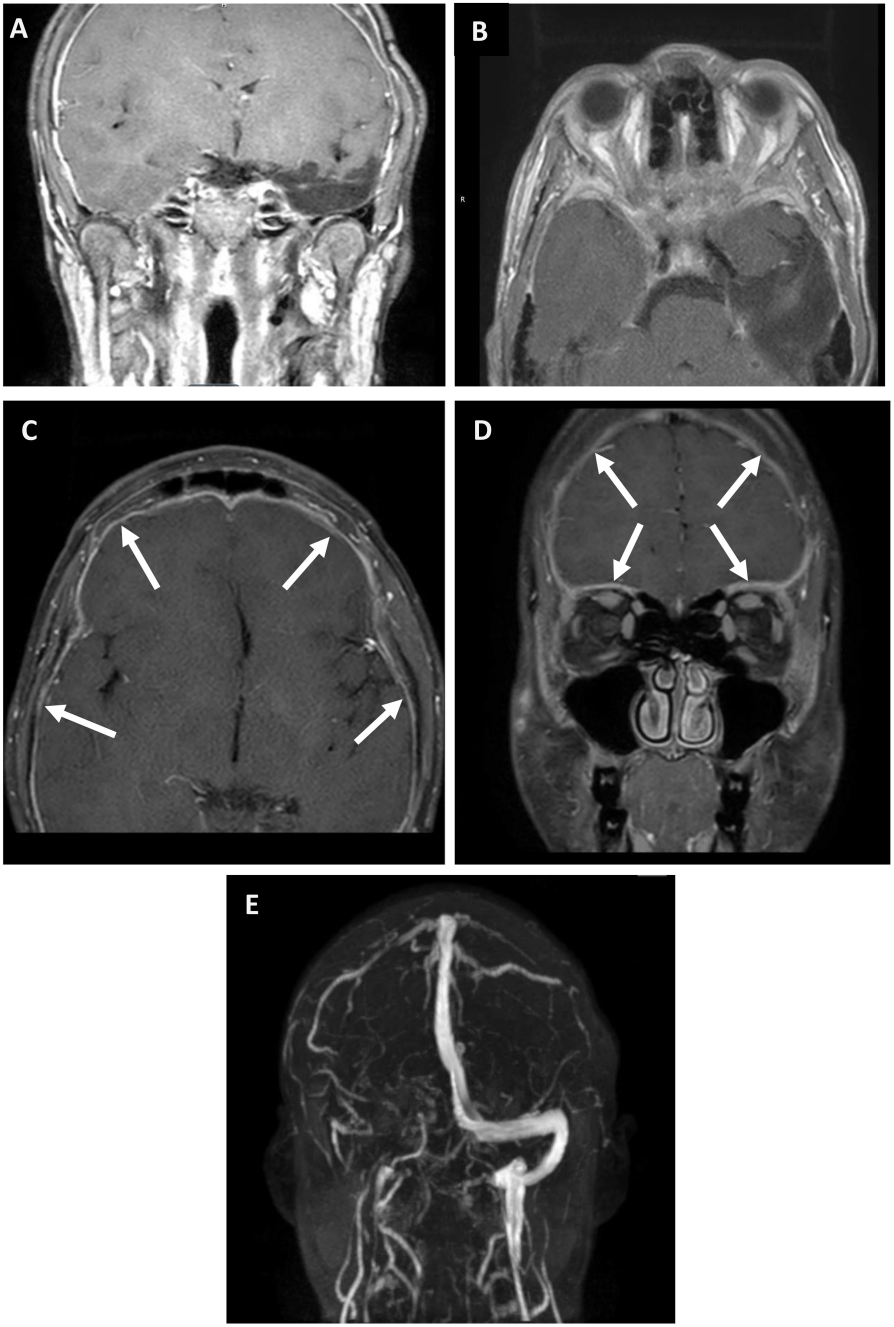
MRI/MRV brain and orbit scans with gadolinium at presentation. **(A–D)**, Axial and coronal T1-weighted postcontrast fat-saturated images, 13 years after a left temporal lobe tumor resection, which demonstrate postoperative changes with diffuse, smooth dural enhancement over the frontal and temporal lobes (arrowheads). **(E)**, Coronal 2D TOF MRV scan showing a congenitally hypoplastic right transverse sinus, and, to a lesser extent, the right sigmoid sinus and the proximal right jugular vein. There was no evidence of a dural sinus thrombosis or findings that were suggestive of an elevated intracranial pressure on neuroimaging.

An emergent brain MRI demonstrated diffuse pachymeningeal thickening and left temporal enhancement, which was initially presumed consistent with the history of tumor resection, but no optic nerve enhancement. Magnetic resonance venography (MRV) showed anatomical variation in the right transverse sinus, the hypoplastic right sigmoid, and the right internal jugular veins (**Figure 2**). Both his right eye visual field defect and his pachymeningeal thickening were thought to be stable findings from his prior tumor resection. He was advised to discontinue acne medications and to start 250 mg of acetazolamide four times daily for (QID) for presumed papilledema secondary to isotretinoin use. Before the initiation of Diamox® therapy, a subsequent second opinion of an adult neuro-ophthalmology evaluation prompted lumbar puncture after the initial presentation showed an opening pressure of 16 cmH_2_O, and, therefore, Diamox therapy was discontinued. With further worsening vision, he was referred to our pediatric neuro-ophthalmology clinic 3 weeks later.

**Figure 3:**
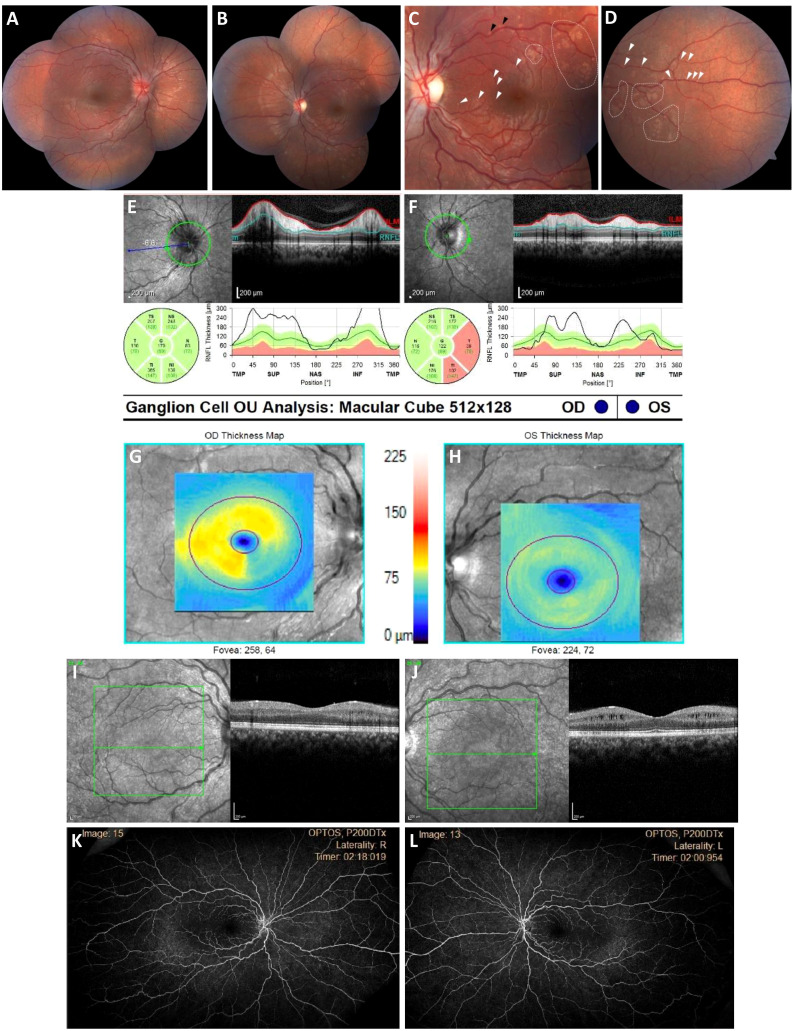
Bilateral optic neuropathy and retinopathy at presentation. **(A, B)**, Widefield fundus color photographs demonstrating an asymmetric right greater than left optic disc edema and numerous patchy RPE abnormalities of the left posterior pole that are consistent with inflammatory lesions. **(C, D)**, High-magnification view of macular subretinal lesions. **(E, F)**, Circumpapillary RNFL thickening is most pronounced in the superior and inferior temporal quadrants OD and superior and inferior quadrants OS. **(G–J)**, Macula OCT with ganglion cell segmentation demonstrating asymmetric ganglion cell layer thinning, and left eye intraretinal cystoid changes of the inner nuclear layer. **(K, L)**, Widefield late-phase fluorescein angiograms showing no peripapillary leakage, vascular leakage, or capillary non-perfusion.

On neuro-ophthalmology examination, he was 20/15 in the right eye and had the ability to detect hand motion in the left eye. His confrontational visual field testing noted a left superior temporal visual defect in the right eye, and a dense central scotoma sparing the peripheral field in the left eye. Automated perimetry redemonstrated the right superior temporal quadrantanopia in the right eye. A dilated fundus examination noted 3+ disc edema in the right eye and mild, pallid disc edema in the left eye. Notably, the retinal examination of the left eye revealed subtle infiltrates cuffing around the retinal vessels along the superior and inferior arcades (**Figure 3**). Optical coherence tomography (OCT) of the circumpapillary retinal nerve fiber layer (RNFL) showed right optic nerve edema and left optic nerve thinning. OCT of the maculae showed asymmetric ganglion cell layer thinning and intraretinal cystoid changes of the inner nuclear layer. A dense scan through the retinal infiltrates to further characterize their location was not obtained. A fluorescein angiogram showed only late staining of the disc in the right eye and no signs of capillary non-perfusion or vascular leakage of either eye. The patient was thought to have an inflammatory optic neuropathy and retinopathy, and a broad systemic workup was initiated. However, the serum studies were all negative, including a complete blood count (CBC), an erythrocyte sedimentation rate (ESR) evaluation, levels of C-reactive protein (CRP), angiotensin-converting enzyme (ACE), and lysozyme, an extended extractable nuclear antigen (ENA) panel (i.e., SSA, SSB, Scl70, RNP, Sm, dsDNA, FANA) test, a QuantiFERON™ test, a rapid plasmin reagin (RPR) card test, a fluorescent treponemal antibody absorption (FTA-Abs) test, tests for herpes simplex virus (HSV), varicella zoster virus (VZV), IgG4, anti-phospholipid Ab, and serum complement, a PCR analysis to detect *Bartonella* and *Toxoplasma*, and HLA-B51 testing, as were serum and cerebrospinal fluid (CSF) studies for anti-myelin oligodendrocyte glycoprotein (MOG) and anti-neuromyelitis optica (NMO) antibodies. The patient and family were offered electroretinography testing, but they deferred due to financial limitations.

**Figure 4:**
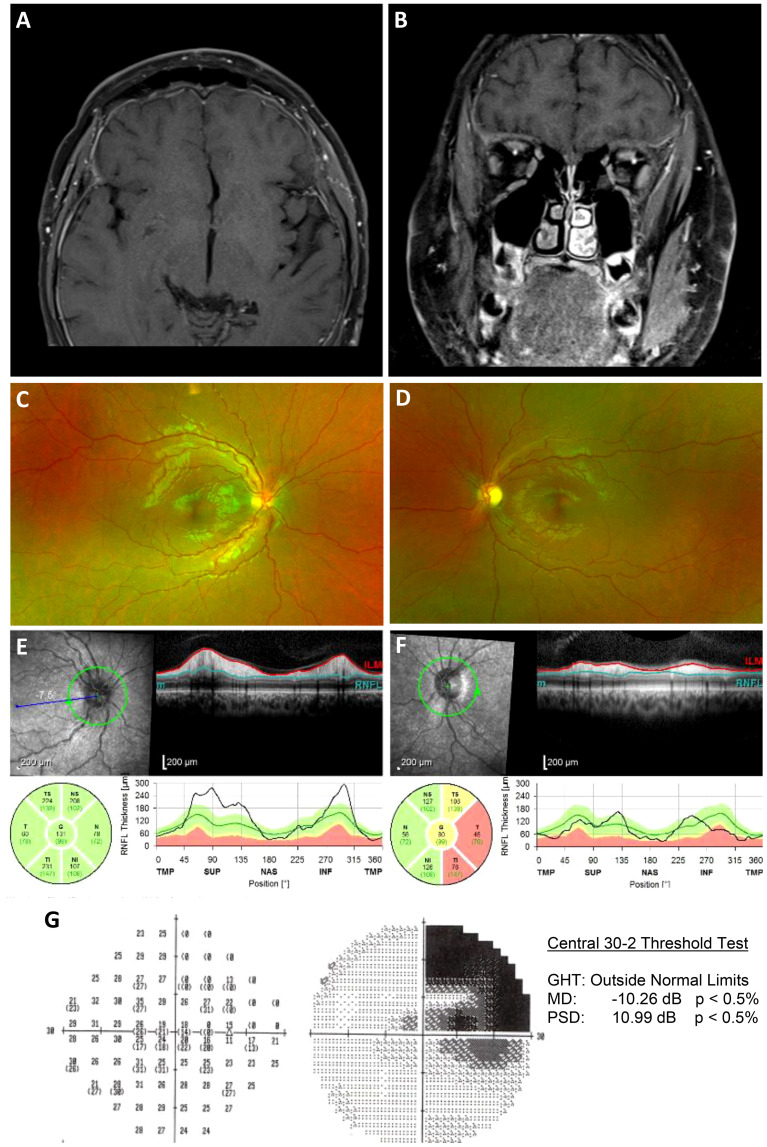
Follow-up post immunosuppressive treatment. **(A, B)**, Axial and coronal T1-weighted postcontrast fat-saturated images after treatment with steroids and IVIG demonstrates a significant interval decrease in dural thickening, with only minimal residual enhancement. **(C, D)**, Widefield fundus photographs demonstrating an improvement in optic nerve edema of both eyes, a marked temporal pallor of the left eye, and resolution of multifocal subretinal infiltrates along the superior and inferior temporal arcades of the left eye. **(E, F)**, Circumpapillary RNFL thickening improved OD and demonstrated inferotemporal thinning OS. **(G)**, Automated perimetry demonstrating an improvement in right superior quadrantanopia, which was previously thought to be due to a ganglioglioma resection.

With a negative workup, an autoimmune retinopathy (AIR) panel was sent to the Casey Eye Institute, Oregon Health and Science University, Portland, OR, USA, with the patient being started on oral prednisone. There was notable stability and improvement at 4 weeks. At 6 weeks, the AIR panel was positive for anti-enolase, anti-recoverin, anti-tubulin, and anti-pyruvate kinase M2, with human immunohistochemical staining of both ganglion cell and photoreceptor layers. He was diagnosed with autoimmune-related retinopathy and optic neuropathy and started on intravenous immunoglobulin (IVIG) therapy. A comprehensive occult malignancy workup, including a PET scan, was negative. At the 3-month follow-up, the patient’s visual acuity improved so that he could count fingers with the left eye, with the right eye demonstrating a significant improvement in the right superior quadrantanopia (**Figure 4**). Repeat fundoscopic examination showed resolution of retinal infiltrates, and repeat MRI scanning showed resolution of the previously noted pachymeningeal thickening.

## Results

In this study, data from our subject and from the five reports of pediatric autoimmune retinopathy and optic neuropathy in the English-language literature to date were accessed. The subjects’ average age was 11.1 years (range 2–15 years). Three were female.

All subjects presented with visual impairment, with one complaining of night blindness, and one complaining of floaters. Four subjects had optic disc edema and two had optic disc pallor in at least one eye at presentation. The retinal findings were described for all six subjects, including bone spicule pigmentation, periphlebitis, mild-peripheral white dots, cystoid macular edema, and perivascular subretinal infiltrates. Vitreous cells were present in two subjects, one of whom complained of floaters.

Electrophysiology testing was described for three subjects, two of whom had extinguished electroretinograms, and one with findings that were consistent with retinal dystrophy. The last subject also had fluorescein angiography demonstrating disc hyperfluorescence, central and peripheral capillary leakage, and mottled hyperfluorescence, but had normal CSF test results and MRI scans. One subject had bilateral peripheral ring scotomas, as detected via Goldmann visual field testing.

An underlying neoplastic disorder was diagnosed in four of the subjects. Two subjects had coincident pineal gland germinomas, one had a remote history of Langerhans cell histiocytosis (LCH), which had been treated 8 years prior, and our subject had a history of left ganglioglioma 10 years prior to his vision loss. The youngest subject was noted to have a preceding viral prodrome. The subject with an electroretinogram (ERG) consistent with retinal dystrophy was eventually diagnosed with retinitis pigmentosa (RP).

The serum autoantibody testing was described for four subjects: the youngest subject with the preceding viral prodrome had antibodies against a 22-kDa antigen; one of the two subjects with pineal gland germinoma had antibodies against a 35-kDa optic nerve-derived antigen; the subject with LCH had antibodies against recoverin and a 62-kDa antigen; and our subject had antibodies against enolase, recoverin, tubulin, and pyruvate kinase M2.

Both subjects with pineal gland germinomas underwent focal radiation, and one also underwent chemotherapy. The subject with a history of LCH underwent treatment with tacrolimus. The subject with RP-like findings underwent treatment with intravitreal triamcinolone acetonide. Our subject was treated with steroid infusions and IVIG.

The treatment outcomes varied. The viral prodrome subject received no treatment and suffered no progression of vision loss. Both of the subjects with pineal gland germinomas and our subject had resolved disc edema and periphlebitis following treatment. The subject with a history of LCH had improved visual acuity and decreased ring scotoma in one eye and stable vision loss in the other eye. The subject with RP-like degeneration had a mild decline in vision, despite having intravitreal therapy.

## Discussion

To our knowledge, our patient is among the youngest cases, found in the literature, presenting with possible autoimmune retinopathy (AIR) and optic neuropathy. Pediatric cases have been reported, but not all would meet the currently accepted criteria of AIR, that is, progressive vision loss, visual field defects, and abnormal rod and/or cone responses on ERG, with or without a diagnosis of cancer (1).

Keltner and Thirkill first reported the case of a 2-year-old female with poor optokinetic responses and moderate optic pallor in both eyes 4 weeks after a varicella zoster infection and tetanus–pertussis–diphtheria inoculation, who, 4 years following the initial presentation, had stable vision loss and showed seropositivity for a 22-kDa retinal antigen (2). The classification of this case as AIR is difficult to reconcile with its non-progressive course and its presentation in close proximity to an antecedent viral illness known to be associated with meningoencephalitis and optic neuritis. Oray et al. reported the case of an 11-year-old female with a family history of retinitis pigmentosa, who presented with subacute bilateral vision loss, vitreous cells, optic disc edema, and cystoid macular edema, and he was later diagnosed with AIR as a secondary complication of retinitis pigmentosa (3). However, we believe this case represents a known complication of inherited retinal dystrophies and is not independently a case of AIR.

Both Chang et al. and Forooghian et al. have reported pediatric cases of retinopathy and optic neuropathy in association with pineal gland germinomas (4, 5). In the case reported by Chang et al., a 14-year-old East Indian boy presented with 2 months of visual and neurologic symptoms, as well as vitreous cells, marked optic nerve head edema, and retinal periphlebitis. He was diagnosed with pineal gland germinoma, and, after focal radiotherapy, his ocular findings resolved. No autoantibody testing was reported. Forooghian et al. reported a 14-year-old male with blurred vision, midbrain pupils, retinal periphlebitis, and bilateral optic disc edema, who was also found to have a pineal gland germinoma. Interestingly, in this case, Western blot analysis against the optic nerve revealed a single band at 35 kDa, which was not re-demonstrated after 6 months of treatment. Both cases differed from ours, however, in that active malignancy co-presented with ocular findings.

Hayashi et al. reported the case of an 11-year-old who underwent chemotherapy and steroid treatment for LCH 8 years prior to the onset of visual disturbance and night blindness with retinitis pigmentosa-like degeneration and small white dots in the mid-periphery of both eyes (6). In addition, the subject’s electroretinogram was extinguished, his Goldmann perimetry showed mid-peripheral ring scotoma in the right eye, and his serum tested positive for anti-recoverin and a 62-kDa antigen. This case best resembled the timeline of our patient, but the optic nerve and retina findings were different.

### Clinical findings: old and new

Among the pediatric cases of autoimmune retinopathies and optic neuropathies, our subject’s retinal findings have not been described before. Although some cases have reported frank retinal periphlebitis (4, 5), most cases of AIR in children, and even adults, present without infiltrative or inflammatory retinal lesions. Although a fluorescein angiogram is useful for highlighting active retinal and choroidal vascular inflammation, subtle or indolent immune-mediated processes may go undetected. Our subject presented with discrete multifocal subretinal lesions along the temporal arcades of the left eye that evaded detection, even from the referring ophthalmologists and a uveitis specialist who excluded inflammatory processes after noting a normal fluorescein angiogram. Our discovery of these lesions, however, prompted antibody testing for autoimmune retinopathy, and, ultimately, treatment with immunosuppression resolved these infiltrates, further supporting an autoimmune mechanism.

This case is also the first to report intracranial neuroinflammatory changes accompanying ocular findings. The presence of diffuse pachymeningeal thickening and enhancement on the presenting MRI was a presumed sequela from the prior surgical resection. However, a more thorough review of prior neuroimaging revealed a surveillance contrast CT scan from 3 years after the initial resection that showed no dural meningeal thickening or enhancement. Moreover, just like the retinal lesions, the pachymeningeal thickening and enhancement also resolved after immunosuppression suggesting the presence of a neuroinflammatory state accompanying the diagnosis of AIR. Interestingly, the initial lumbar puncture did not demonstrate an elevated opening pressure and CSF analysis did not demonstrate inflammation. Collectively, abnormal neuroimaging, a normal opening pressure on lumbar puncture, asymmetric optic nerve edema/pallor, and a left visual field with central vision loss sparing the peripheral vision all lowered the concern for idiopathic intracranial hypertension as a potential confounding diagnosis.

### Paraneoplastic versus non-paraneoplastic AIR

Our subject’s remote history of malignancy brings to the attention the classification of autoimmune retinopathy and optic neuropathy as paraneoplastic versus non-paraneoplastic. In adults, paraneoplastic autoimmune retinopathy (pAIR) is the more prevalent of the two, and the most commonly implicated malignancies are, in order: breast, melanoma, lung, prostate, gynecological, colon, lymphoma, thyroid, and bladder (1). Neurologic malignancies are associated with only 2% of pAIR in adults (1). The association between malignancies and pAIR is not known in children, as the incidence of AIR is rare. This limited case series (**Table 1**) notes an incidence of malignancies in 66% (*n* = 4/6) of pediatric cases of AIR. Thus, a plausible hypothesis in support of pediatric pAIR is that our subject had a mixed tumor comprised partly of glial tissue, which is associated with a three- to fivefold increase in serum recoverin levels (7, 8). Our subject may have developed humoral immunity against his primary tumor, harbored anti-recoverin antibodies in his serum, and after some inciting event that compromised his blood–brain barrier, experienced the propagation of anti-recoverin-mediated ganglion cell layer and photoreceptor apoptosis (9). The other anti-retinal antibodies may then represent the epiphenomenon of retinal degeneration. Although tumor pathology supports an anti-recoverin-mediated pAIR diagnosis, the other clinical features of this case do not (10). The asymmetric and subacute presentation many years after diagnosis of the malignancy, as well as the responsiveness to steroid immunosuppression, do not fit the typical course of anti-recoverin-mediated pAIR (10).

**Table 1:** Review of clinical presentation of pediatric autoimmune retinopathies and optic neuropathies.

Author (Year)	Age, Sex	Chief Complaint	Clinical Findings	Work-up	Primary Disorder	Serum Ab(s)	Treatment	Outcome
Keltner and Thirkill (1999) (2)	2y, F	Vision loss	Narrow blood vessels, bone spicules, moderate optic pallor OU	ERG: extinguished OU	“Viral syndrome”	22 kDa antigen	–	Stable VA loss
Chang et al (1999) (4)	14y, M	Visual impairment, floaters	Upgaze paresis, vitreous cells, retinal periphlebitis, bilateral optic disc edema	N/a	Pineal gland germinoma	N/A	Focal radiation	Resolved disc edema and periphlebitis
Forooghian et al (2006) (5)	14y, M	Blurred vision	Midbrain pupils, retinal periphlebitis, bilateral optic disc edema	N/a	Pineal gland germinoma	35 kDa antigen (optic nerve)	Focal radiation, chemo- therapy	Resolved disc edema and periphlebitis
Hayashi et al (2007) (6)	11y, F	Visual disturbance, night blindness	RP-like degeneration, small white dots in the mid-periphery	ERG: extinguished, GVF: mid-peripheral ring scotoma OU	LCH (8 yrs prior)	Anti- recoverin and 62 kDa antigen	Tacrolimus	Improved VA, decreased ring scotoma OD; stable VA loss OS
Oray et al (2013) (3)	11y, F	Vision loss	Vitreous cells, optic disc edema, cystoid macular edema	CSF: normal; MRI: normal; IVFA: disc hyperfluorescence, central and peripheral capillary leakage and mottled hyper-fluorescence; SDOCT: CME, SRF, and schisis-like cavity; ERG: consistent with retinal dystrophy	RP	N/A	Intravitreal triamcinolone acetonide	Mild decline in VA; eventual emergence of subtle peripheral pigmentary atrophy
Varma et al (2023)	15y, M	Subacute vision loss OS, then OD	Disc edema OD, disc pallor OS, subtle RPE lesions along vessels OS; right superior quadrantanopia OD; dense central scotoma OS	MRI: pachymeningeal enhancement; CSF: normal, including opening pressure; HVF: right superior quadrantanopia OD; OCT: CME and severe GCL loss OS; sectoral GCL loss OD; IVFA: normal;	Left temporal Ganglio- glioma (12 years prior)	Anti-enolase, anti- recoverin, anti- tubulin, anti- pyruvate kinase M2; human IHC staining of photoreceptor and ganglion cell layers	Steroids, IVIG	Improved VF OD, stable vision loss OS; resolved retinal findings and pachymeningeal enhancement

ERG, electroretinography; kDa, kilodalton; VA, visual acuity; RP, retinitis pigmentosa; GVF, Goldmann visual field; LCH, Langerhans cell histiocytosis; IVFA, intravenous fluorescein angiography; SDOCT, spectral domain optical coherence tomography; CME, cystoid macular edema; SRF, subretinal fluid; RPE, retinal pigment epithelium; HVF, Humphrey visual field; GCL, ganglion cell layer; IVIG, intravenous immunoglobulin

Alternatively, our subject’s clinical course more closely resembled anti-enolase-mediated AIR, and, therefore, suggests the possibility of an independent non-paraneoplastic autoimmune mechanism (10). In this scenario our subject’s immune response was initiated against a microbial insult, and subsequently developed cross-reactivity between pathogen and host glycolytic enzymes, such as enolase, tubulin, and pyruvate kinase M2 (9). The anti-recoverin antibodies, in this scenario, may then represent the epiphenomenon of retinal degeneration.

Finally, the combination of subacute retinal infiltrates, macular edema, and the rapid response to anti-inflammatory therapy still matches the tempo of an inflammatory etiology that may not yet be clearly elucidated or described. Frequent surveillance of this patient looking for any additional systemic symptoms as well as other ocular findings will be necessary ultimately to better characterize this entity and to determine the potential need for maintenance immunosuppression in the long term.

### Role of retinal autoantibodies

The diagnostic utility of autoantibodies in cases of vision loss accompanying subtle or atypical ocular findings remains a topic of debate. Are autoantibodies formed from prior sensitization to intrinsic or extrinsic immune challenge destroying host retinal tissue or is the degeneration of host retinal tissue from another mechanism generating autoantibodies against liberated retinal antigens?

In our case, two of the four detected autoantibodies closely correlated with the clinical presentations previously attributed to each other in other reports. Anti-enolase antibodies are associated with subacute vision loss, nyctalopia, and retinal atrophy. Anti-tubulin antibodies are associated with central visual field loss (1). Our subject presented exactly with subacute, central-predominant vision, and visual field loss of the left eye with evolving central visual loss of the right eye. This correlation was further supported by the positive immunohistochemical staining of the human retina targeting the photoreceptor and ganglion cell layers by our subject’s serum. Clinically, this was mirrored by OCT ganglion layer dropout (or thinning) in our patient.

Among the other cases of pediatric autoimmune retinopathy and optic neuropathy that describe autoantibody testing, only the report by Hayashi et al. shows similar clinicopathologic correlation. The authors recovered a 62-kDa protein from their subject’s serum, which may now be classified as heat shock protein 60 (HSP-60), an autoantibody associated with sudden-onset visual acuity loss and attenuated ERG response. The 22-kDa antigen, described by Keltner and Thirkill, and the 35-kDa antigen, reported by Forooghian et al., do not have an identified function in the phototransduction pathway.

Several studies have demonstrated the poor specificity of current antiretinal testing and its limited clinical application for suspected AIR diagnosis (11, 12). Chen et al. noted in a cross-sectional study that 93% of patients without AIR tested positive for multiple anti-retinal antibodies; however, none of these patients had anti-recoverin antibodies. Our case is consistent with these studies and only supports the clinical utility of autoantibody testing specifically in the setting of clinicopathologic presentation from either the previously described phenomena of autoantibody-mediated retinopathy and optic neuropathy, or the new clinical findings we describe. Chen et al. also noted, however, that 64% of patients without a diagnosis of AIR had alpha-enolase antibodies. Confirmatory immunohistochemical staining in human retina was also present in 12 out of 14 (86%) samples in patients without an AIR diagnosis. Therefore, neither the presence of the antibody in our patient, nor IHC staining of human retina by the patient’s serum is diagnostic; therefore, autoantibody testing should not be weighted as sole diagnostic criteria.

Overall, pediatric autoimmune retinopathy and optic neuropathy is a rare disease that can present with a broad spectrum of active and indolent clinical findings. In pediatric patients presenting with (1) a history of prior or active oncology, (2) clinical findings of progressive retinopathy or optic neuropathy, and (3) a negative inflammatory workup with suggestive ERG abnormalities, autoimmune retinopathy, and optic neuropathy should be considered in the differential, taking into account cautious interpretation of anti-retinal antibody testing.

## Data availability statement

The original contributions presented in the study are included in the article/supplementary material. Further inquiries can be directed to the corresponding author.

## Ethics statement

Written informed consent was obtained from the individual(s), and minor(s)’ legal guardian/next of kin, for the publication of any potentially identifiable images or data included in this article.

## Author contributions

HV: Writing – original draft. KZ: Writing – original draft, Writing – review & editing. VS: Writing – original draft, Writing – review & editing.

## References

[1] Adamus G Champaigne R Yang S. Occurrence of major anti-retinal autoantibodies associated with paraneoplastic autoimmune retinopathy. Clin Immunol (2020) 210:108317. doi: 10.1016/j.clim.2019.108317 31770612 PMC6989367

[2] Keltner JL Thirkill CE. The 22-kDa antigen in optic nerve and retinal diseases. J Neuroophthalmol. (1999) 19:71–83. doi: 10.1097/00041327-199906000-00001 10380127

[3] Oray M Kir N Tuncer S Onal S Tugal-Tutkun I. Autoimmune retinopathies: a report of 3 cases. Ocul Immunol Inflamm (2013) 21:424–33. doi: 10.3109/09273948.2013.799215 23730997

[4] Chang CW Hay D Chang TS Nguyen R Lyons CJ. Retinal periphlebitis in a patient with pineal germinoma. Arch Ophthalmol (1999) 117:1434–6. 10532463

[5] Forooghian F Chew HF Muni RH Adamus G Drake JM Buncic JR. Paraneoplastic optic disc oedema and retinal periphlebitis associated with pineal germinoma. Br J Ophthalmol (2007) 91:985–6. doi: 10.1136/bjo.2006.112193 PMC1955653 17576721

[6] Hayashi M Hatsukawa Y Yasui M Yanagihara I Ohguro H Fujikado T. Cancer-associated retinopathy in a child with Langerhans cell histiocytosis. Jpn J Ophthalmol (2007) 51:393–6. doi: 10.1007/s10384-007-0457-y 17926119

[7] Sampath P Ce W Sungarian A Cortez S Alderson L Stopa EG. Cerebrospinal fluid (vascular endothelial growth factor) and serologic (recoverin) tumor markers for Malignant glioma. Cancer Control. (2004) 11:174–80. doi: 10.1177/107327480401100305 15153841

[8] Manley P Li X Turner C Chi S Zimmerman MA Chordas C. A prospective, blinded analysis of A-PROTEIN (recoverin or CAR protein) levels in pediatric patients with central nervous system tumors. Pediatr Blood Cancer. (2009) 53:343–7. doi: 10.1002/pbc.22017 19422022

[9] Adamus G. Are anti-retinal autoantibodies a cause or a consequence of retinal degeneration in autoimmune retinopathies? Front Immunol (2018) 9:765. doi: 10.3389/fimmu.2018.00765 29713325 PMC5911469

[10] Adamus G Ren G Weleber RG. Autoantibodies against retinal proteins in paraneoplastic and autoimmune retinopathy. BMC Ophthalmol (2004) 4:5. doi: 10.1186/1471-2415-4-5 15180904 PMC446200

[11] Faez S Loewenstein J Sobrin L. Concordance of antiretinal antibody testing results between laboratories in autoimmune retinopathy. JAMA Ophthalmol (2013) 131:113–5. doi: 10.1001/jamaophthalmol.2013.574 23307224

[12] Chen JJ McKeon A Greenwood TM Flanagan EP Bhatti MT Dubey D. Clinical utility of antiretinal antibody testing. JAMA Ophthalmol (2021) 139:658–62. doi: 10.1001/jamaophthalmol.2021.0651 PMC8063133 33885761

